# Numerical study on the supersonic gas-solid two-phase injection mechanism of needle-free syringe

**DOI:** 10.1371/journal.pone.0322571

**Published:** 2025-05-29

**Authors:** Xiao Mo, Yang Xiao, Kai-Xiong Qing, Feng Zhang, Hongshi Yu

**Affiliations:** 1 Faculty of Information Engineering and Automation, Kunming University of Science and Technology, Kunming, Yunnan, China; 2 Department of Cardiovascular Surgery, The First Affiliated Hospital of Kunming Medical University, Kunming, Yunnan, China,; 3 State Key Laboratory of Complex Nonferrous Metal Resources Clean Utilization, Kunming University of Science and Technology, Kunming, Yunnan, China; Donghua University, CHINA

## Abstract

Supersonic gas-solid injection technology finds extensive use in drug particle delivery systems. However, the combined impact of particle diameter and mass flow rate on the delivery efficiency remain insufficiently explored. Within the Euler-Lagrange framework, this study utilizes the discrete phase method (DPM) for the numerical simulation of supersonic gas-particle flow in a needle-free injector. After validating the model’s accuracy with experiment results, further investigations were conducted into the influences of particle size and mass flow rate on particle behavior and flow field properties. The results indicate that the impact of larger particles on the compressible structure is stronger, while higher mass flow rate absorbs greater energy from the gas phase, reducing the gas expansion capacity, which results in lower velocity, Mach number, and higher temperature. The jet core zone is approximately *x/X *= 0.3 in length. Outside core zone, the gas velocity rapidly decays and temperature rises sharply. Within the jet core zone, drug particles are accelerated and cooled, while beyond core zone, they decelerate and heat up. The strongest inter-phase interactions occur primarily in the nozzle expansion area and the jet core zone. Smaller particles reach maximum velocity upstream. This implies that in designing needle-free injectors, the nozzle-to-skin distance must match the drug particle diameter to achieve maximum penetration effectiveness. Furthermore, the particle temperature decreases with smaller sizes. As the particle diameter rises from 10 μm to 100 μm, the minimum temperatures of the particles are 145 K and 264 K, respectively, indicating the need to match the particle diameter with the minimum temperature at which the drug particles remain effective. Additionally, higher mass flow rate doses reduce injection velocity and penetration ability, necessitating the rational control of the administered dose range. These results offer significant theoretical guidance for the design and improvement of needle-free injection.

## 1 Introduction

Needle-free injection devices are widely used for the delivery of various drugs (such as insulin) and vaccines [1,2].The basic principle involves the use of supersonic airflow generated by a Laval nozzle to accelerate drug particles, enabling them to penetrate the skin. This technology offers advantages such as avoiding pain and infection, and enhancing drug absorption [3–5]. The injection process of needle-free injectors is a classic example of supersonic gas-particle flow. Unlike supersonic single-phase gas flow, the particle phase in gas-particle flow results in more complex behavior, especially under high Mach number (*Ma*) conditions, where gas compressibility, shock waves, and expansion waves considerably complicate both gas dynamics and particle kinetics. Furthermore, in specific applications such as supersonic needle-free injectors, the diversity and complexity of drug delivery parameters further complicate the insights into supersonic gas-particle flow. Therefore, a more profound comprehension of supersonic gas-particle flow behavior is crucial for improving the performance of such devices.

So far, a series of studies have focused on exploring the dynamics of supersonic gas- particle flow. For instance, Kendall et al. [6] controlled the particle impact speed using contoured shock tube (CST) and light gas gun (LGG), and precisely measured the final position and particle penetration depth within the skin using optical microscopy and image analysis software. The experimental results provided standards for selecting the optimal particle parameters and impact velocity. Zhang et al. [7] studied the airflow pressure and particle velocity distribution through particle tracking velocimetry (PTV) and pressure measurements. The study showed that the nozzle outlet flow occurs in two distinct stages: the initial phase and the stable phase. In the initial stage, the particle count fluctuated greatly, while in the steady-steady stage, the particle count tended to become uniform. Michinaka et al. [8] explored the influence of different injection distances on particle delivery efficiency. The findings indicated that maximum delivery efficiency occurred when the injector was positioned 3 mm from the skin surface, with about 90% of the dose successfully stayed in the skin, when the distance was reduced to 1 mm, the retention rate dropped to about 60%. Uchida et al. [9] injected particles into the mouse skin under different helium pressures (100, 200, and 300 psi). The results showed that as the helium pressure increased, the depth and quantity of microsphere penetration into the skin significantly increased, with the best penetration effect observed at 300 psi. Kendall et al. [10] used particle image velocimetry (PIV) to measure the particle speed distribution at the CST exit. The experimental findings indicated that the CST can transport particles to the targeted skin area at a uniform speed of ± 4% during the steady state, thereby achieving precise drug delivery. The aforementioned experimental studies have deepened the understanding of these systems and provided valuable insights. However, the experimental methods failed to capture crucial particle-scale information, such as inter-phase interactions, which limits further understanding of supersonic gas-solid injection.

In recent years, advancements in computational hardware and numerical techniques have established computational fluid dynamics (CFD) as a crucial approach to studying supersonic gas-particle flow. Research methods in this field are primarily categorized into two frameworks: Euler-Lagrange and Euler-Euler [11–13]. The two differ in how the discrete phase is handled. The solid phase is viewed as a continuous phase in the Euler-Euler model, reducing computational requirements, but it cannot track individual particle trajectories or capture particle-scale information. To address these issues, the Euler-Lagrange model was introduced, treating particles as discrete phases for tracking, allowing for more accurate particle-scale information. Considering the handling of the discrete phase and inter-phase interactions, a series of Euler-Lagrange-based models have been developed, such as discrete phase method (DPM), have been widely applied in analyzing supersonic gas-particle flows.

For instance, Liu et al. [14] employed DPM to simulate the transient gas-particle flow within a Laval nozzle, finding that high pressure drop and a high nozzle throat area ratio caused excessive expansion at the nozzle outlet, resulting in flow separation and shock wave phenomena, which aggravated pressure and velocity field unevenness, thereby affecting particle flow uniformity. Rasel et al. [15] used DPM to simulate and analyze the interaction of gas and drug particles within the CST system. They combined numerical simulations and experimental validation to find that higher rupture pressures increased particle velocity, and the CST system could achieve a uniform spatial distribution of particle velocity, with pressure and output velocity controlled by adjusting the diaphragm thickness. Zhang et al. [16] used DPM to simulate gas-particle flow, describing the gas flow using unsteady Reynolds-Averaged Navier-Stokes (RANS) equations. and accounting for the impact of temperature on viscosity. The results showed that the boundary layer significantly influenced particle speed. Particle speeds decreased near the pipe wall, while higher velocities occurred in the uniform velocity region. Liu et al. [17] simulated the supersonic gas-particle flow through convergent-divergent supersonic nozzle (CDSN) and CST nozzles. The results showed that the CDSN system caused uneven particle velocity distribution and unstable flow due to oblique shock waves, whereas the CST system exhibited uniform acceleration with an average speed of 699 m/s and a standard deviation of only 4.7 m/s, demonstrating consistent and controllable velocity distribution. Rasel et al. [18] used CFD and DPM to simulate the CST drug delivery system, employing the resistance correlation model to predict particle behavior in unsteady flow. The results showed that the CST system significantly accelerated particles. Additionally, a rise in density lowered the particle outlet velocity, but the distribution uniformity persisted. Zhang et al. [19] adopted DPM to research the behavior of polydisperse particles in supersonic nozzle gas jets. The findings demonstrate that increasing the particle injection speed usually increases the average particle velocity while reducing the velocity heterogeneity and dispersion. However, when the injection particle velocity exceeds 100 m/s, the opposite trend occurs.

Although previous studies have revealed the basic characteristics of supersonic gas-particle flow to some extent, how to precisely control the drug dosage and particle diameter for optimal injection effects remains an important issue that has not been fully addressed. To fill this research gap, this study uses the DPM to conduct an in-depth analysis of supersonic gas-particle flow dynamics within the Laval nozzle. The focus of the investigation includes the compressibility of the continuous phase, particle acceleration characteristics, temperature distribution, as well as the interplay between the gas and particles. Particular attention is also given to the role and mechanisms of particle size and drug delivery rate in influencing the overall behavior of supersonic gas-powder flow, providing theoretical support for optimizing injection performance.

## 2 Mathematical approaches

### 2.1 Gas phase governing equations

During the process of flow acceleration from subsonic to supersonic speeds, the gas expands extremely rapidly, while solid particles, due to inertia or heat transfer lag, cannot synchronously respond to the changes in the gas state, resulting in strong relaxation effects. To address these strong relaxation effects, this study has created a numerical model grounded in the Euler-Lagrange framework. Specifically, the equations of conservation for mass, momentum, and energy are presented as follows:


$$\frac{\partial }{\partial t}\left(\alpha_{g}\rho_{g}\right)+\nabla \cdot \left(\alpha_{g}\rho_{g}u_{g}\right)=0$$ (1)

$$\frac{\partial }{\partial t}\left(\alpha_{g}\rho_{g}u_{g}\right)+\nabla \cdot \left(\alpha_{g}\rho_{g}u_{g}u_{g}\right)=-\alpha_{g}\nabla P+\nabla \cdot \left(\alpha_{g}\tau_{eff}\right)+\alpha_{g}\rho_{g}g-\frac{1}{V_{cell}}F_{gp}$$ (2)

$$\frac{\partial \left(\alpha_{g}\rho_{g}C_{p,g}T_{g}\right)}{\partial t}+\nabla \cdot \left(\alpha_{g}\rho_{g}u_{g}C_{p,g}T_{g}\right)=\alpha_{g}\nabla \cdot \left(\tau_{eff}\cdot u_{g}\right)+\alpha_{g}\lambda_{eff}\nabla^{2}T_{g}-\frac{1}{V_{cell}}\sum Q_{conv}$$ (3)

In the equation, *α*_*g*_ represents the gas volume fraction; *ρ*_*g*_ represents the gas density, determined by applying the ideal gas law; *λ*_*eff*_ stands for the effective thermal conductivity accounting for the turbulence effect. *P* stands for the static pressure, ***u***_*g*_ and *T*_*g*_ signifies the speed and temperature of the gas, respectively. ***F***_*gp*_ represents the force exerted by gas on particles, including all contributions in equation (11). It should be noted that the interphase coupling of momentum occurs only between the gas phase and the particle phase and is not related to gravity. Additionally, *Q*_*conv*_ represents heat transfer between the two phases. where *C*_*p,g*_ represents the specific heat capacity of the gas. ***τ***_*eff*_ represents the effective stress tensor, including Newtonian viscous stress tensor and Reynolds stress tensor, defined as follows:


$$\begin{array}{cc} & \tau_{eff}=\left[\mu_{g}\left(\nabla u_{g}+\nabla u_{g}^{T}\right)-\frac{2}{3}\mu_{g}\delta_{i,j}\left(\nabla \cdot u_{g}\right)\right]+\\ & \left[\mu_{t}(\nabla u_{g}+\nabla u_{g}^{T})-\frac{2}{3}\mu_{t}\delta_{i,j}\left(\nabla \cdot u_{g}\right)-\frac{2}{3}\rho_{g}k\delta_{i,j}\right] \end{array}$$ (4)

Here, *μ*_*g*_ represents the gas phase’s dynamic viscosity and *μ*_*t*_ denotes the turbulent viscosity; $\nabla {\text{u}}_{\text{g}}$ denotes the gradient of the gas velocity vector, and $\nabla {\text{u}}_{\text{g}}^{\text{T}}$ refers to its transpose. To accurately model the turbulent behavior of the gas phase, this research employs a realizable *k-ε* turbulence model formulated using the RANS method, coupled with a standard wall function to decrease grid resolution in the boundary layer. This turbulence model has been widely validated through numerical studies on supersonic gas-particle dynamics [20–22]. Below are the equations that govern turbulent kinetic energy (*k*) and its dissipation rate (*ε*) [23]:


$$\mathrm{\backslash [}\frac{\partial }{\partial t}\left(\rho_{g}k\right)+\frac{\partial }{\partial x_{j}}\left(\rho_{g}ku_{j}\right)=\frac{\partial }{\partial x_{j}}\left[\left(\mu_{g}+\frac{\mu_{t}}{\sigma_{k}}\right)\frac{\partial }{\partial x_{j}}\right]+G_{k}-\rho_{g}\varepsilon -Y_{m}\mathrm{\backslash ]}$$ (5)

$$\mathrm{\backslash [}\frac{\partial }{\partial t}\left(\rho_{g}\varepsilon \right)+\frac{\partial }{\partial x_{j}}\left(\rho_{g}\varepsilon u_{j}\right)=\frac{\partial }{\partial x_{j}}\left[\left(\mu_{g}+\frac{\mu_{t}}{\sigma_{\varepsilon }}\right)\frac{\partial \varepsilon }{\partial x_{j}}\right]+\rho_{g}C_{1}S\varepsilon -\rho_{g}C_{2}\frac{\varepsilon^{2}}{k+\sqrt{\nu \varepsilon }}\mathrm{\backslash ]}$$ (6)

Here, *u*_*j*_ represents the velocity component of the fluid in the *x*_*j*_ direction; *G*_*k*_ denotes the turbulent kinetic energy generation resulting from the mean velocity gradient; and *Y*_*m*_ is the turbulence dissipation due to buoyancy. *σ*_*k*_ and *σ*_*ε*_, which represent the turbulent Prandtl numbers for *k* and *ε*, are set at 1.0 and 1.2, respectively. *C*_*2*_ denotes an empirical constant, with a value of 1.9. The calculation formula for *C*_*1*_ is:


$$C_{1}=\max\left(0.43,\frac{\varphi }{\varphi +5}\right)$$ (7)

$$\varphi =S\frac{k}{\varepsilon }$$ (8)

$$S=\sqrt{2SijSij}$$ (9)

### 2.2 Solid phase governing equations

To precisely characterize the particle motion states, this study establishes the dynamic equation of discrete phase under the Lagrangian framework, following Newton’s second law, with the goal of tracking particle motion. The summation of all forces influencing the particle is represented on the right-hand side, which collectively influence its motion state. The dynamic equation is as follows [24]:


$$m_{p}\frac{du_{p}}{dt}=F_{gp}+F_{g}$$ (10)

$$F_{gp}=F_{d}+F_{l}+F_{m}+F_{Pr\text{e}}$$ (11)

***F***_*d*_ stands for the drag force; ***F***_*l*_ denotes the lift force; ***F***_*g*_ represents gravitational force. It should be noted that gravity is not a dominant factor in the present research. However, to ensure the completeness of the model, the gravity is still taken into consideration. ***F***_*m*_ represents the virtual mass force; ***F***_*d*_ represents the pressure gradient force. The calculation formula for drag force ***F***_*d*_ is:


$$F_{d}=m_{p}\frac{u_{g}-u_{p}}{\tau_{r}}$$ (12)

Here, *τ*_*r*_ is the relaxation time [25]. The calculation formula is:


$$\tau_{r}=\frac{\rho_{p}d_{p}^{2}}{18\mu_{g}}\frac{24}{C_{\text{D}}Re_{p}}$$ (13)

Here, *ρ*_*p*_ describes the particle’s density, whereas *d*_*p*_ indicates its diameter, *μ*_*g*_ denotes the gas viscosity, and *Re*_*p*_ represents the particle Reynolds number.


$$Re_{p}=\frac{\rho_{g}d_{p}\left|u_{p}-u_{g}\right|}{\mu_{g}}$$ (14)

*C*_*D*_ represents the drag coefficient, and its calculation formula is:


$$C_{D}=a_{1}+\frac{a_{2}}{Re_{p}}+\frac{a_{3}}{Re_{p}^{2}}$$ (15)

This formula is derived from the drag coefficient model for spherical particles proposed by Morsi and Alexander [26], where constants *a*_*1*_, *a*_*2*_, and *a*_*3*_ are selected based on the relative *Re*_*p*_. Considering the significant influence of high Mach numbers on the particle drag coefficient, *C*_*D*_ has been modified according to reference [27]. Specifically, the method for drag coefficient correction is as follows: First, construct a bivariate interpolation table of drag coefficients for different *Re*_*p*_ and *Ma*, according to Fig 10.15 of the reference [27]. Then, the velocity, density, viscosity of the cell where a specific particle located in can be acquired and the corresponding *Re*_*p*_ of this particle can be calculated. Combined with the *Ma* of this cell, the drag coefficient can be finally obtained through two-dimensional linear interpolation using the interpolation table. This approach can more accurately solve the gas-solid interaction under supersonic conditions, effectively addressing the problem of strong relaxation. Besides, the rarefaction effect also has a non-negligible effect on the drag coefficient [28], which can be ignored in the current work by evaluating the threshold of Knudsen number. The lift calculation formula in Eq. (11) is represented as follows:

**Fig 1:**
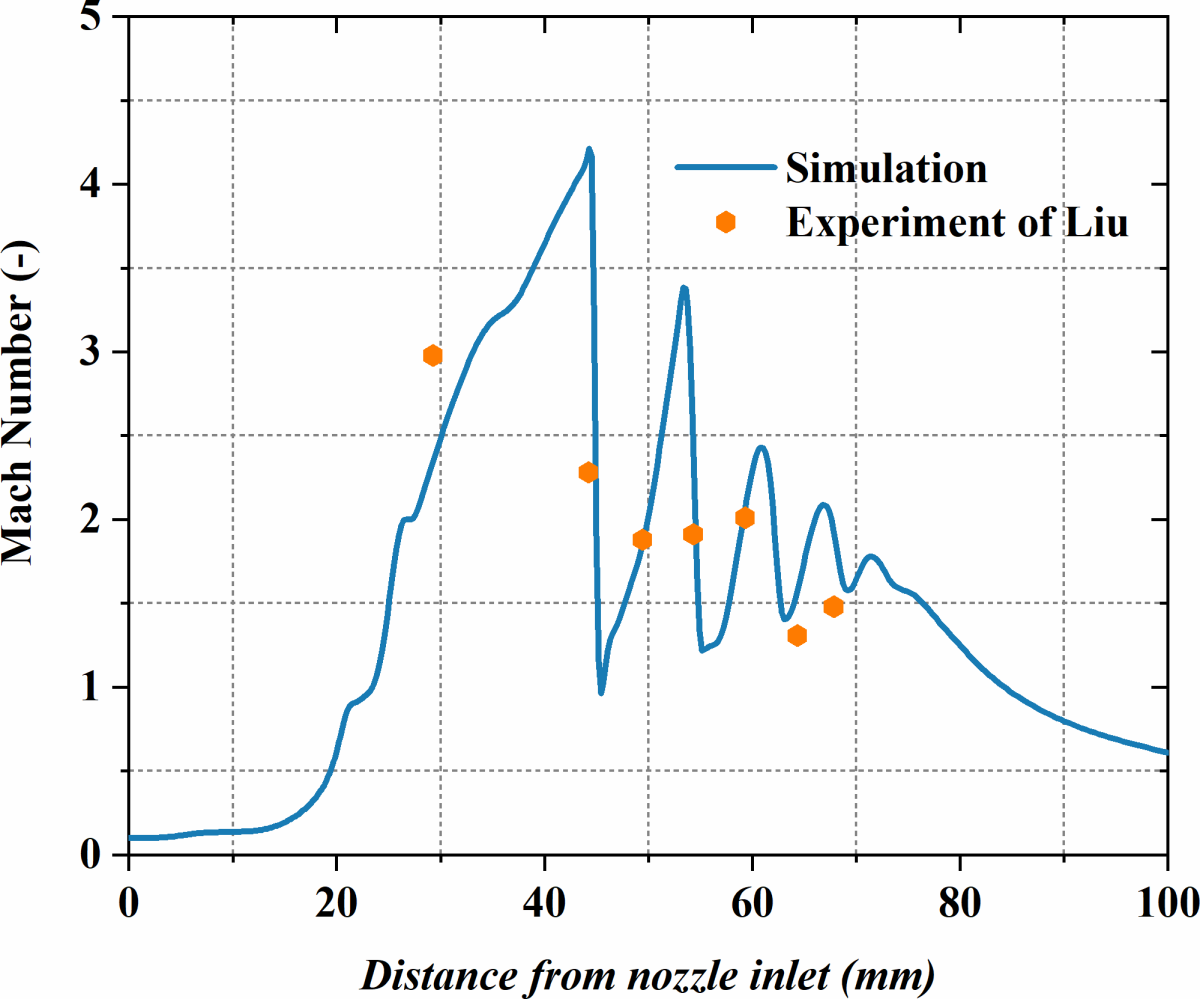
Distribution of Mach number along the centerline of the nozzle: Comparison between current simulation results and experimental data from Liu [17].

**Fig 2:**
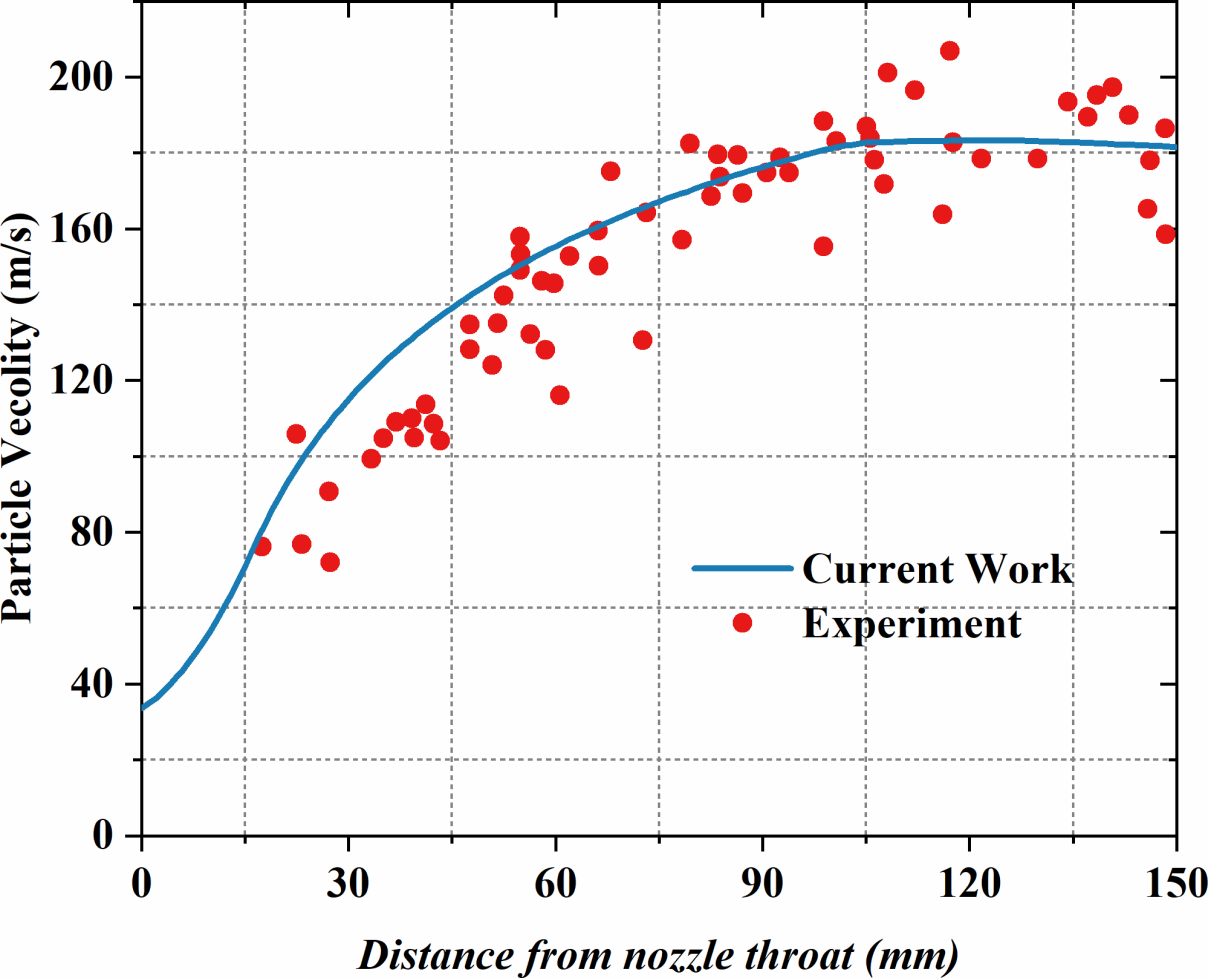
Analysis of particle speed comparisons between numerical simulation and Okuda [31] experimental results.

**Fig 3:**
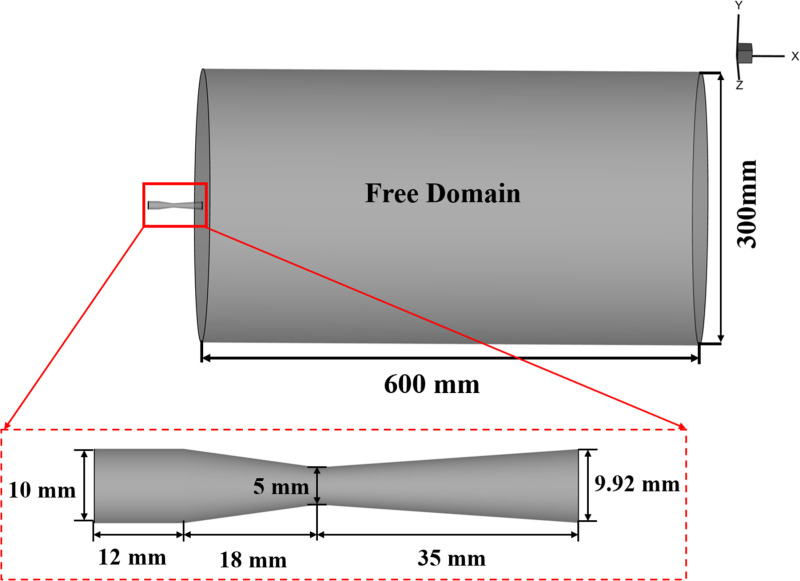
Schematic view of the 3D computational domain and Laval nozzle structural parameters.

**Fig 4:**
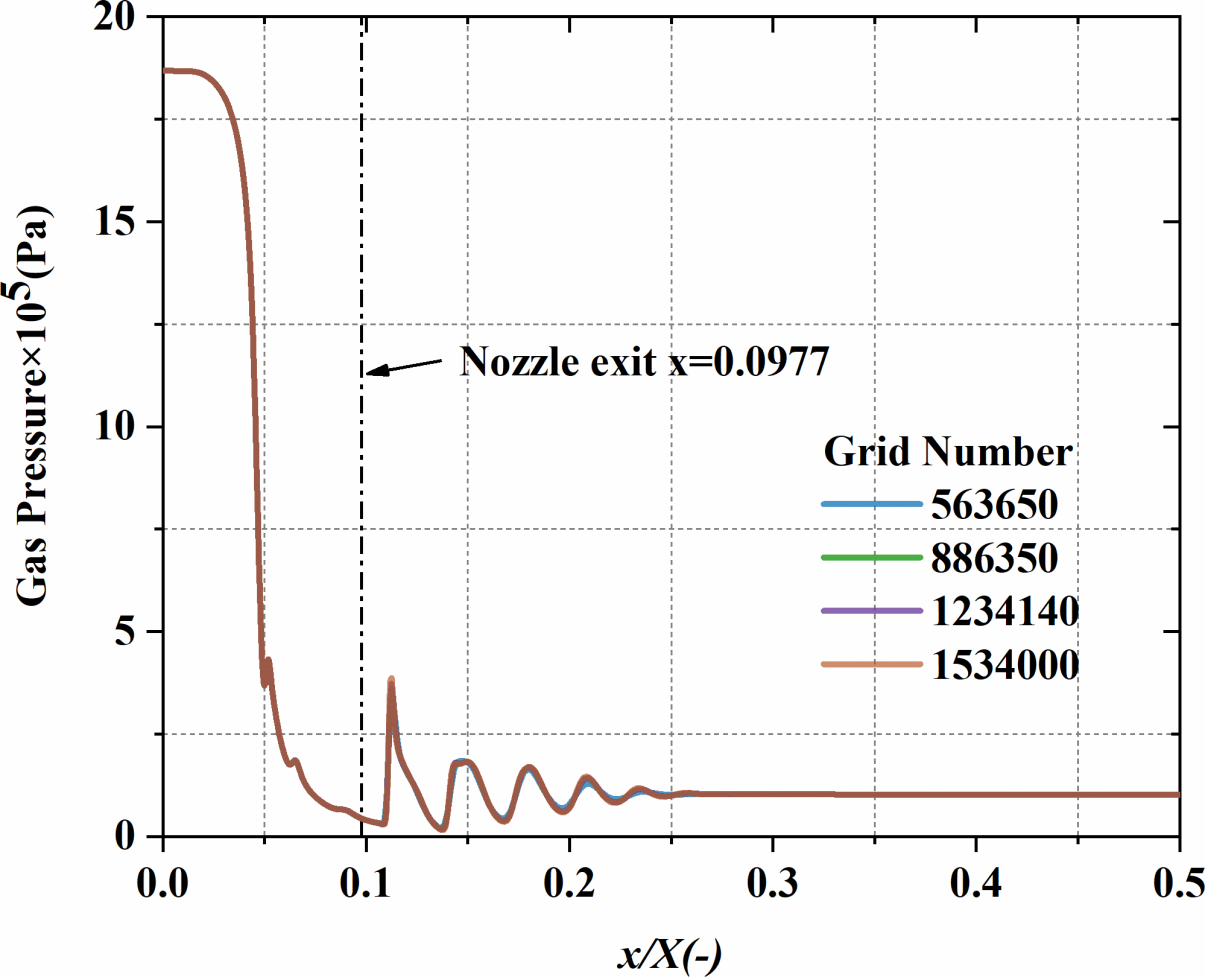
Axial gas-phase pressure variation along the centerline of the computational domain under various grid settings.

**Fig 5:**
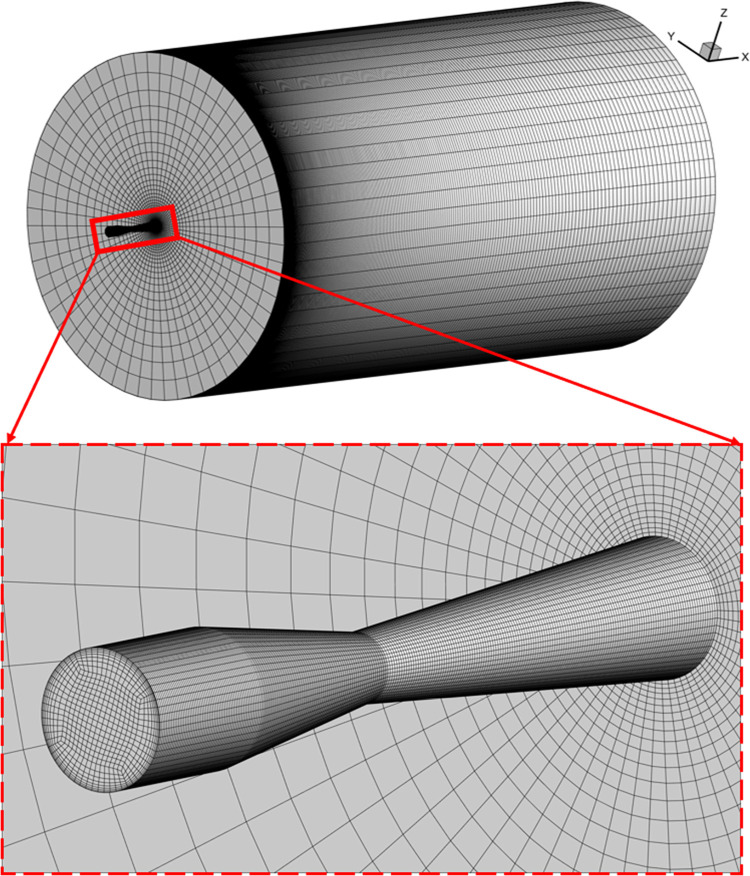
Mesh distribution of the 3D computational domain and nozzle.

**Fig 6:**
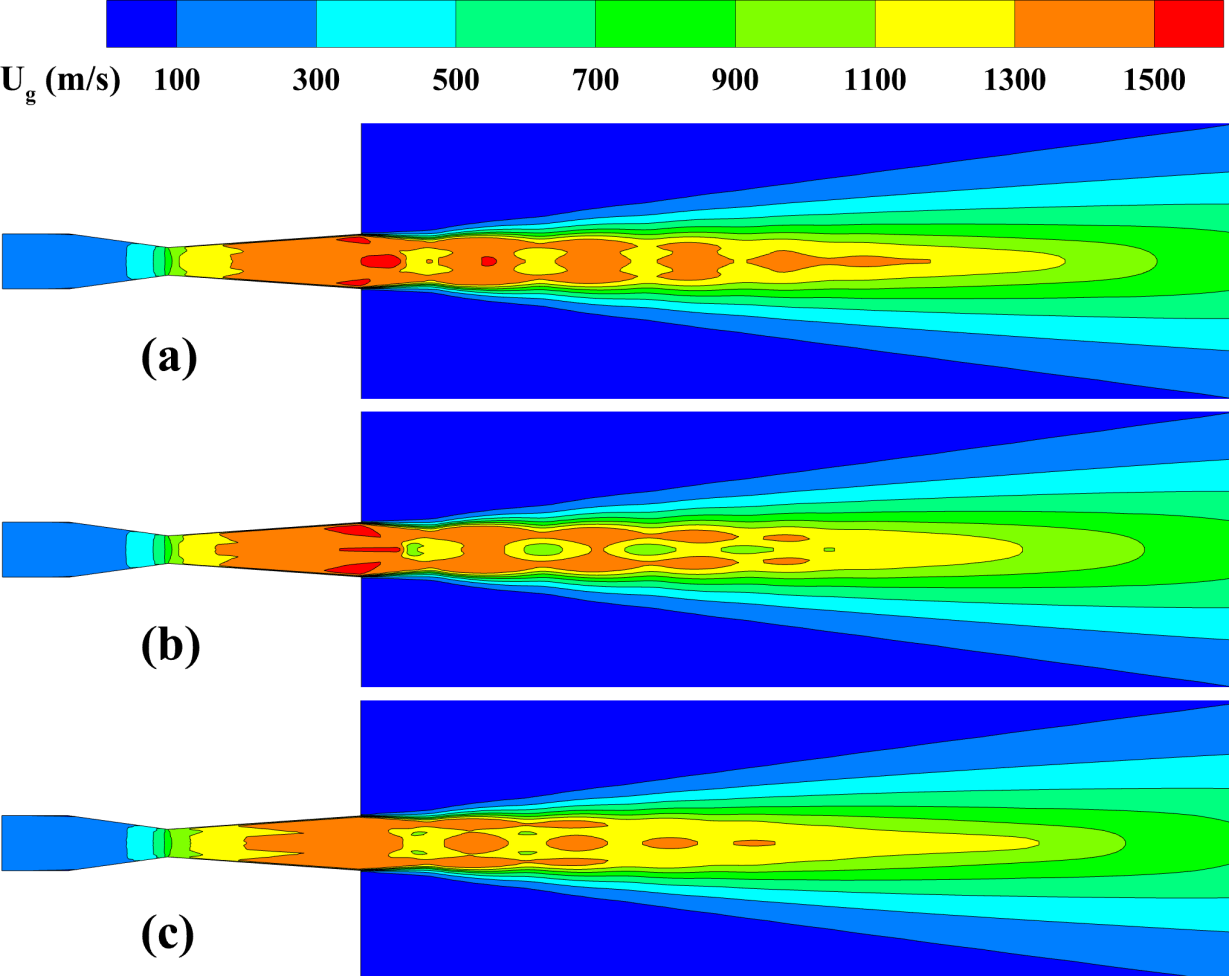
Gas speed distribution on the central plane. **(a) *d***_***p***_** = 30 μm, *Q***_***p***_** = 5 × 10**^**-3**^ **kg/s; (b) *d***_***p***_** = 100 μm, *Q***_***p***_** = 5 × 10**^**-3**^ **kg/s; (c) *d***_***p***_** = 30 μm, *Q***_***p***_** = 1 × 10**^**-2**^ **kg/s.**

**Fig 7:**
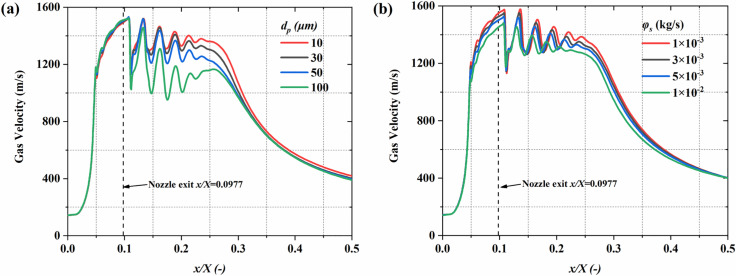
The influence of particle parameters on the time-averaged gas speed distribution along the system centerline. (a) particle diameter; (b) mass flow rate.

**Fig 8:**
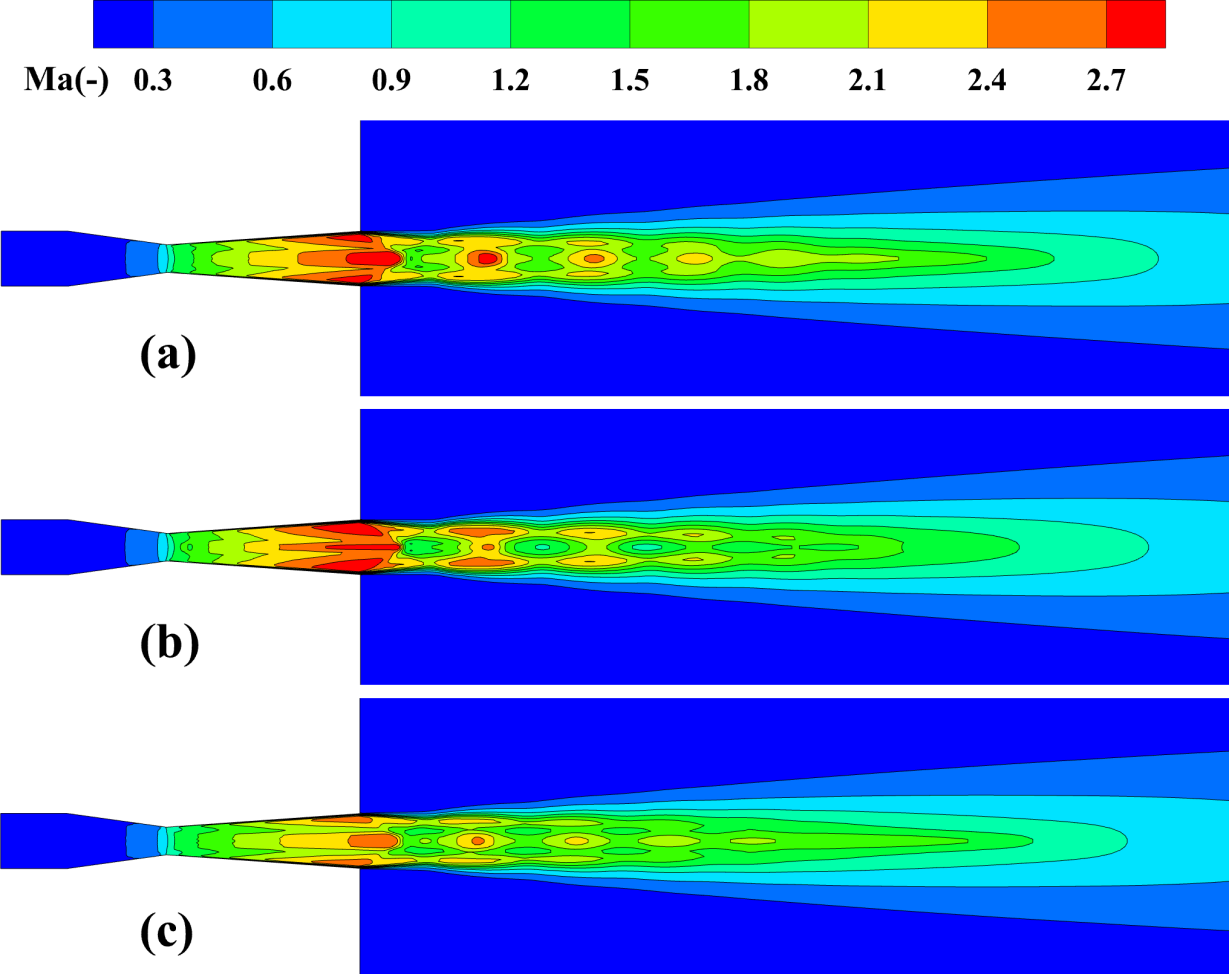
*Ma* distribution on the central plane. **(a) *d***_***p***_** = 30 μm, *Q***_***p***_** = 5 × 10**^**-3**^ **kg/s; (b) *d***_***p***_** = 100 μm, *Q***_***p***_** = 5 × 10**^**-3**^ **kg/s; (c) *d***_***p***_** = 30 μm, *Q***_***p***_** = 1 × 10**^**-2**^ **kg/s.**

**Fig 9:**
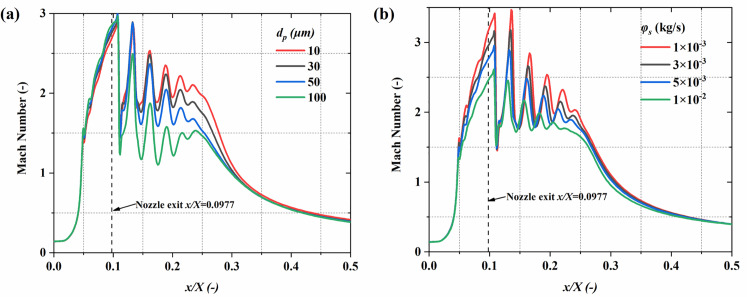
The impact of particle parameters on the time-averaged *Ma* distribution along the system centerline. (a) particle diameter; (b) mass flow rate.


$$F_{l}=C_{L}\frac{\pi d_{p}^{4}}{4}\frac{\rho_{g}|u_{p}-u_{g}|\left(u_{p}-u_{g}\right)}{2}$$ (16)

Here, the lift coefficient *C*_*L*_ is 0.5, and $\pi d_{p}^{4}$ represents the characteristic geometric factor based on the particle diameter. It is important to highlight that the current research does not account for particle rotation and the determination of *C*_*L*_ is according to the particle radial dispersion. For more details, please refer to our previous research [29]. The gravitational force ***F***_*g*_ is defined as:


$$F_{g}=m_{p}\frac{g\left(\rho_{p}-\rho_{g}\right)}{\rho_{p}}$$ (17)

The virtual mass force ***F***_*m*_ is defined as:


$$F_{m}=0.5m_{p}\frac{\rho_{g}}{\rho_{p}}\left(u_{p}\nabla u_{g}-\frac{du_{p}}{dt}\right)$$ (18)

The pressure gradient force ***F***_*pre*_ is defined as:


$$F_{pre}=m_{p}\frac{\rho_{g}}{\rho_{p}}u_{g}\nabla u_{g}$$ (19)

This study analyzes the exchange of heat between particles and the ambient environment during motion. Given the sparsity of particles, while conductive heat exchange between solid phase is neglected, only convective heat transfer *Q*_*conv*_ and radiation heat transfer *Q*_*rad*_ are considered. Based on this, the particle temperature governing equation is expressed as:


$$m_{p}C_{p,p}\frac{dT_{p}}{dt}={\;\;\;\Phi \;\;\;}_{p}$$ (20)

$${\;\;\;\Phi \;\;\;}_{p}=Q_{conv}+Q_{rad}$$ (21)

Here, *C*_*p,p*_ represents the particle’s specific heat capacity; *Φ*_*p*_ denotes the thermal power of the particle. *Q*_*conv*_ and *Q*_*rad*_ representing convective and radiative heat transfer respectively, are defined as:


$$Q_{conv}=\frac{\lambda_{g}Nu_{p}}{d_{p}}A_{p}(T_{g}-T_{p})$$ (22)

$$Q_{radi}=\sigma \varepsilon_{p}A_{p}\left(T_{b,local}^{4}-T_{p}^{4}\right)$$ (23)

Here, *σ*, the Stefan-Boltzmann constant, equals 5.67 × 10^-8^ *W/*(*m*^*2*^*K*^*4*^), where *ε*_*p*_ and *A*_*p*_ denote the particle’s emissivity and surface area, respectively. *T*_*b,local*_ represents the wall surface temperature; and *λ*_*g*_ signifies the gas thermal conductivity. *Nu*_*p*_ denotes the particle Nusselt number, a dimensionless heat transfer parameter, computed using the following equation:


$$Nu_{p}=2+0.6Re_{P}^{1/2}Pr^{1/3}$$ (24)

Here, *Pr* denotes the Prandtl number. Finally, it is important to highlight that in the current research, the maximum particle volume fraction of all cases is 5.3 × 10^-5^, which is much lower than the threshold of 10^-3^ according to the reference [30]. Therefore, in the numerical model, particle collisions are disregarded.

## 3 Verification of the model and numerical setup

This research focuses on the axial acceleration of drug particles in supersonic airflow. The complex compressible flow structures and gas-solid interactions at high Mach numbers significantly increase the difficulty of predicting particle behavior. To address this, two model validations were conducted, confirming that the current model can precisely forecast the compressible flow structures of pure gas-phase flow as well as the axial acceleration characteristics of the particles.

### 3.1 Verification Ⅰ

This validation aims to evaluate the predictive accuracy of the established model for compressible flow characteristics in pure gas-phase flow. By comparing the mass flow rate and outlet *Ma* from the simulation results with the theoretical values derived from the isentropic theory for pure gas, the model’s accuracy has been validated. The relevant calculation formulas are as follows:


$$\frac{A_{e}}{A_{throat}}=\frac{1}{Ma}{\left(\left(\frac{2}{k+1}\mathrm{\backslash rightleft}(1+\left(\frac{k-1}{2}\right)Ma^{2}\right)\right)}^{\frac{k+1}{2(k-I)}}$$ (25)

$$Q_{m}=\frac{A_{throat}P_{t}}{\sqrt{T_{t}}}\sqrt{\frac{\gamma }{R}}{\left(\frac{\gamma +1}{2}\right)}^{-\frac{\gamma +1}{2(\gamma -1)}}$$ (26)

In the equation, *A*_*e*_ denotes the exit area, and *A*_*throat*_ denotes the throat area, *R* represents the gas constant, *k* denotes the specific heat ratio, *P*_*t*_ is the inlet pressure, *T*_*t*_ denotes the gas temperature. By comparing the theoretical calculation values with the numerical results, the theoretical exit *Ma* is 3.4, and the mass flow rate is 0.03432 kg/s. The outlet *Ma* and mass flow rate forecasted by the model are 3.27 and 0.03423 kg/s, with deviations of 3.784% and 0.252%, respectively. In addition, the author’s previous research has also validated the accuracy of the model for pure gas-phase Laval nozzles, referring to the literature [29] for details. The findings suggest that this model achieves a high level of accuracy in predicting compressible flow behavior properties of the gas phase within the Laval nozzle.

For a more thorough assessment of the current model’s predictive accuracy regarding compressible flow structures, this study reconstructs and simulates the Laval nozzle experiment conducted by Liu [17]. In the experiment, helium is utilized as the gas phase, nozzle inlet pressure is 1.1 MPa and the exit pressure is 0.1 MPa. Fig 1 depicts the axial *Ma* distribution at the system’s centerline. It is evident that the numerical data of the current model shows a good match with the experimental measurements, demonstrating that the model can precisely forecast the compressible flow characteristics of the gas, thus validating its reliability and accuracy under complex flow conditions.

### 3.2 Verification Ⅱ

The motion of particles in supersonic gas-particle flow is mainly influenced by drag forces. However, due to the significant variations in *Re*_*p*_ and *Ma* over a wide range, the impact on the drag coefficient is highly complex, which increases the difficulty in accurately predicting axial particle movement. To address this, the study reconstructs and simulates the Laval nozzle No. 1 used in the experiment by Okuda and Choi [31] for numerical validation. In the experiment, the gas phase is chosen to be air, and its density is calculated based on the ideal gas law. The inlet has a total pressure set to 0.392 MPa and a temperature of 293 K, spherical polystyrene particles (0.545 mm in diameter and 1050 kg/m³ in density) are released at an initial speed of 30 m/s. The particle mass loading is set to 0.33, indicating the ratio between the powder feeding rate and gas mass flow rate, ensuring consistency with the experimental conditions. In Fig 2, the current model’s prediction of the particle speed distribution along the nozzle centerline is compared with the experimental results of Okuda and Choi. The findings suggest that the current model’s prediction of the mean particle speed closely matches the experimental data, effectively capturing the axial motion behavior of the particles.

### 3.3 Numerical setup

Fig 3 provides a schematic view of the 3D computational domain in this study, and Table 1 presents the model parameters of the Laval nozzle as well as the physical characteristics of gas and particle, the gas is helium, and the particle consists of polystyrene. The inlet total pressure for the gas phase is configured to be 1.9 MPa, and the nozzle wall is assumed to satisfy no-slip and adiabatic boundary conditions. The standard wall function is employed to solve the flow within the boundary layer. The outlet boundary conditions are specified to match the ambient temperature and static pressure, which are 300 K and 0.1 MPa, respectively. To prevent the particles from escaping the computational domain, at the inlet, particles are released with a starting speed of 1 m/s, and the initial temperature is set at 300 K. To explore the impacts of particle size and mass flow rate on supersonic gas-particle flow, particles measuring 10, 30, 50, and 100 μm in diameter are chosen, and the mass flow rates are 1 × 10^-3^, 3 × 10^-3^, 5 × 10^-3^, and 1 × 10^-2^ kg/s, respectively. Under baseline operating conditions, the particle diameter is 30 μm, and the mass flow rate is 5 × 10^-3^ kg/s. The time step for both the fluid and particles is set to 1 × 10^-6^ s. Adopting such a small-time step ensures that the interphase interactions can be updated in a timely manner, effectively addressing the strong relaxation phenomena characteristic of supersonic gas-solid two-phase flows.

**Table 1:** Nozzle design parameters and gas-solid properties.

Category	Parameter	Value
Nozzle design parameters	Inlet diameter (mm)	10
	Convergent section length (mm)	18
	Throat diameter (mm)	5
	Divergent section length (mm)	35
	Exit diameter (mm)	9.92
Gas phase properties (helium)	Density (kg/m³)	Ideal gas state equation
	Thermal conductivity (W/m·K)	Varies with temperature (piecewise linear)
	Dynamic viscosity (Pa·s)	Varies with temperature (piecewise linear)
	Specific heat capacity (J/kg·K)	5193
Solid phase properties (polystyrene)	Specific heat capacity (J/kg·K)	1300
	Density (kg/m³)	1050
	Particle inlet velocity (m/s)	1
	Diameter (μm)	10, 30, 50, 100
	Particle mass flow rate (kg/s)	1 × 10^-3^, 3 × 10^-3^, 5 × 10^-3^, 1 × 10^-2^

To verify that the computational results remain unaffected by grid size, four different grid sizes were used to carry out a grid independence analysis: 563,650, 886,350, 1,234,140, and 1,534,000 cells. Fig 4 illustrates the gas pressure distribution along the computational domain’s centerline in the axial direction for different grid size. A final grid resolution of 1,234,140 cells was chosen to balance computational accuracy and resource consumption. The grid distribution is shown in Fig 5.

## 4 Results and discussions

### 4.1 Gas properties

#### 4.1.1 Gas velocity.

Fig 6 illustrates the central plane gas speed distribution under different particle sizes and mass flow rate conditions. The gas velocity rises sharply beyond the nozzle throat, reaches a peak at the exit, and then gradually decays. In the lower region of the nozzle flow, the gas speed shows alternating low-velocity zones, indicating the formation of a typical Mach disk. This phenomenon arises due to the combined effects of gas expansion and compression waves. The particle diameter demonstrates a substantial impact on the gas speed distribution. As illustrated in Fig 6b, when the particle diameter increases to 100 μm, the gas speed in the downstream area decreases significantly. This occurs because of the enhanced interaction between larger particles and the gas, leading to a more pronounced transfer of gas kinetic energy to the particle. Additionally, mass flow rate also impacts the velocity. As shown in Fig 6c, at 1 × 10^-2^ kg/s, the range of the high-velocity region is significantly shortened compared to Fig 6a. The increase in particle number results in more gas kinetic energy being transferred to the particles, weakening the gas’s expansion ability, and causing a more rapid decay of velocity downstream. Fig 7 further quantitatively analyzes the variation of gas velocity along the system’s centerline under different particle conditions. Regardless of the changes in particle size or mass flow rate, the gas velocity always exhibits a typical “peak—fluctuation—decay” pattern. However, different particle conditions significantly affect the specific values and fluctuation ranges of the gas speed. Specifically, at 100 μm in diameter, the gas velocity fluctuates between 951 m/s and 1458 m/s. When the particle size decreases from 10 μm, the gas speed fluctuation range increases to 1160 m/s to 1519 m/s. Smaller particles, with lower inertia, cause relatively limited energy loss, which allows the gas velocity to remain at higher levels. The variation in mass flow rate also significantly affects the gas speed fluctuation range. At 1 × 10^-3^ kg/s, the gas velocity fluctuates between 1131 m/s and 1578 m/s, when increased to 1 × 10^-2^ kg/s, the fluctuation range narrows to 1174 m/s to 1481 m/s. The phenomenon suggests that the rise in particle number results in a more significant transfer of gas kinetic energy to the particles, causing a decline in the peak gas speed and a drop in the fluctuation amplitude.

#### 4.1.2 Mach number.

Fig 8 depicts the *Ma* distribution on the central plane under varying particle size and mass flow rate conditions, clearly revealing the alternating distribution of expansion and compression waves located downstream from the outlet. This periodic fluctuation arises from the combined effects of expansion and compression in the supersonic jet, and is significantly modulated by the particle characteristics. In Fig 8b, as the particle diameter increases, the particle inertia increases, leading to enhanced momentum exchange with the gas, causing the disturbance intensity in the flow field to increase further. The fluctuation amplitude of the gas *Ma* increases significantly, and the distribution becomes more uneven. As shown in Fig 8c, at 1 × 10^-2^ kg/s, the *Ma* peak at the outlet reduces significantly. Fig 9 quantitatively illustrates the impact of particle diameter and mass flow rate on the gas Mach number. The results show that smaller particle diameters significantly increase both the *Ma* peak and the flow field stability. Specifically, with a particle size of 10 μm, the *Ma* peak is 2.85, with a minimum value of 1.5. However, when the particle diameter increases to 100 μm, the peak decreases to 2.49 Ma, and the minimum value drops to 1.1 Ma. Smaller particles, due to their lower inertia, have weaker interactions with the gas, reducing the dissipation of gas kinetic energy and allowing the *Ma* peak to remain higher. At 1 × 10^-3^ kg/s, the gas *Ma* peak reaches 3.45 at *x/X *= 0.136, And when it rises to 1 × 10^-2^ kg/s, the *Ma* peak decreases to 2.45 and shifts slightly upstream to *x/X *= 0.13. This highlights that under high mass flow conditions, the gas-solid momentum exchange further reduces the energy transfer efficiency of the gas in the jet core region, causing the *Ma* peak to move closer to the nozzle exit while accelerating the axial decay of the gas speed. It is important to highlight that the current research remains within the range of low particle loading. Although the increase in the mass flow rate of drug particles results in a reduction in the *Ma* of the flow field, the distinct Mach disk structure is still preserved. In contrast, when the particle loading rate is significantly high, the Mach disk structure would be notably disrupted [29,32,33].

#### 4.1.3 Gas temperature.

**Fig 10:**
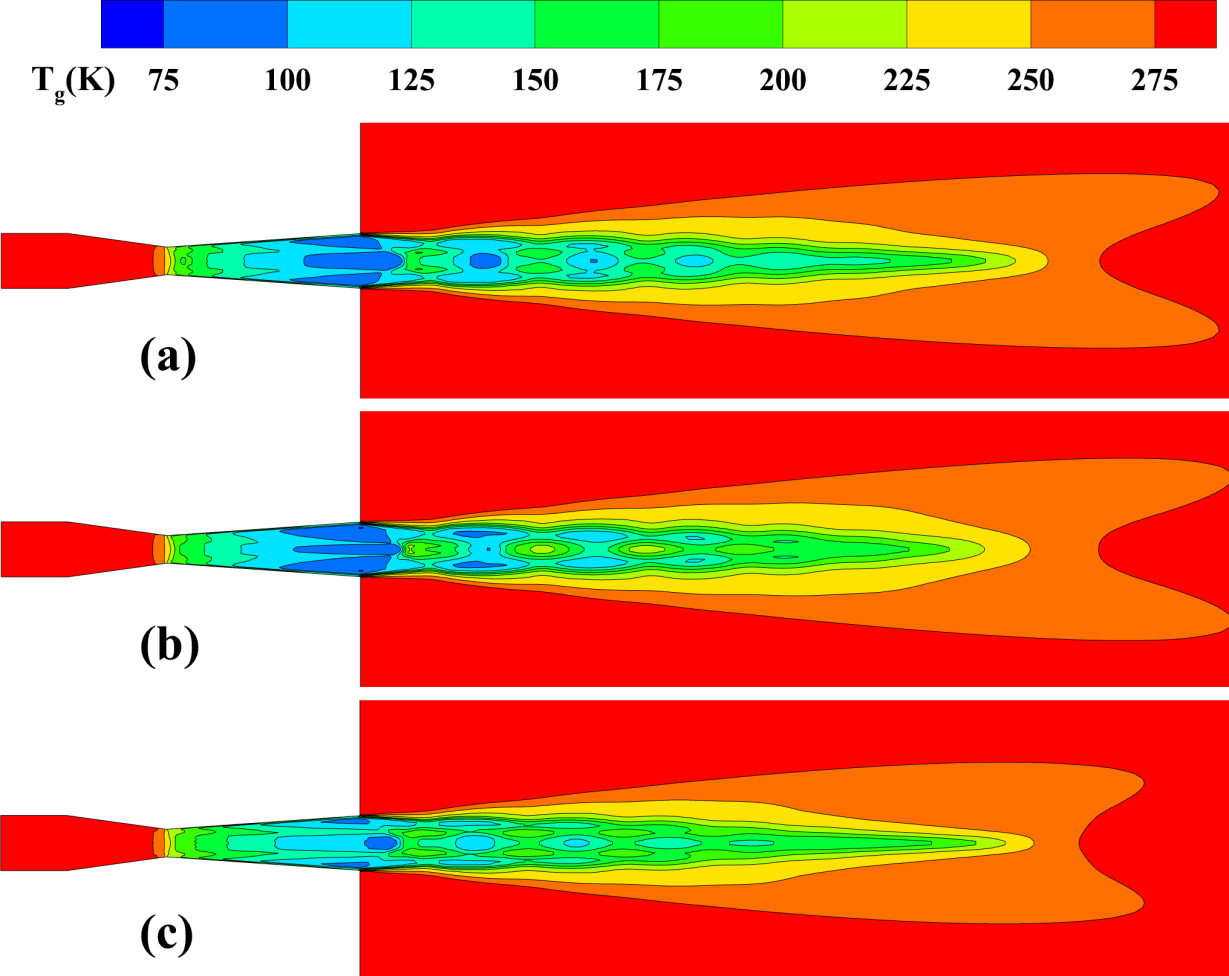
Gas temperature distribution on the central plane. **(a) *d***_***p***_** = 30 μm, *Q***_***p***_** = 5 × 10**^**–3**^ **kg/s; (b) *d***_***p***_** = 100 μm, *Q***_***p***_** = 5 × 10**^**–3**^ **kg/s; (c) *d***_***p***_** = 30 μm, *Q***_***p***_** = 1 × 10**^**–2**^ **kg/s.**

**Fig 11:**
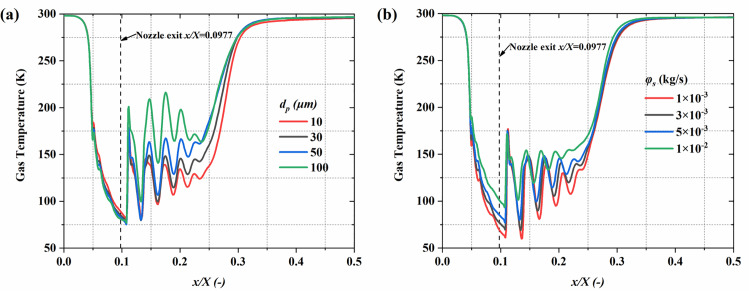
The influence of particle parameters on the time-averaged gas temperature distribution along the system centerline. (a) particle diameter; (b) mass flow rate.

Fig 10 illustrates the central plane gas temperature distribution under different particle size and mass flow rate conditions. It reveals the axial decreasing trend of gas temperature in the nozzle, reflecting the energy conversion mechanism during expansion. However, the particle diameter and mass flow rate play a significant role in affecting the temperature distribution, resulting in noticeable differences in temperature field uniformity and the extent of high-temperature regions under varying conditions. Under conditions with larger particle diameters, the higher particle mass and inertia reduce heat exchange efficiency with the gas phase, leading to increased temperature distribution non-uniformity, particularly in the downstream region where local high temperatures are more prominent. In contrast, higher mass flow rate conditions significantly increase particle concentration, which limits gas phase expansion and weakens the cooling effect, resulting in an overall increase in gas phase temperature while shortening the low-temperature region. Fig 11 provides a quantitative analysis of how particle size and mass flow rate affect the gas temperature distribution. The findings indicate that larger particle diameters significantly increase the gas temperature. At 100 μm in diameter, the maximum gas temperature is 216 K, whereas with a particle diameter of 10 μm, the maximum temperature is only 174 K. This is attributed to the higher surface-to-volume ratio of smaller particles, leading to stronger heat exchange with the gas phase, absorbing more heat and thus lowering the gas temperature. From 1 × 10^-3^ kg/s to 1 × 10^-2^ kg/s, gas phase expansion is further restricted, and the cooling effect is significantly weakened, causing the minimum gas temperature to rise from 60 K to 93 K.

#### 4.1.4 Gas total pressure.

**Fig 12:**
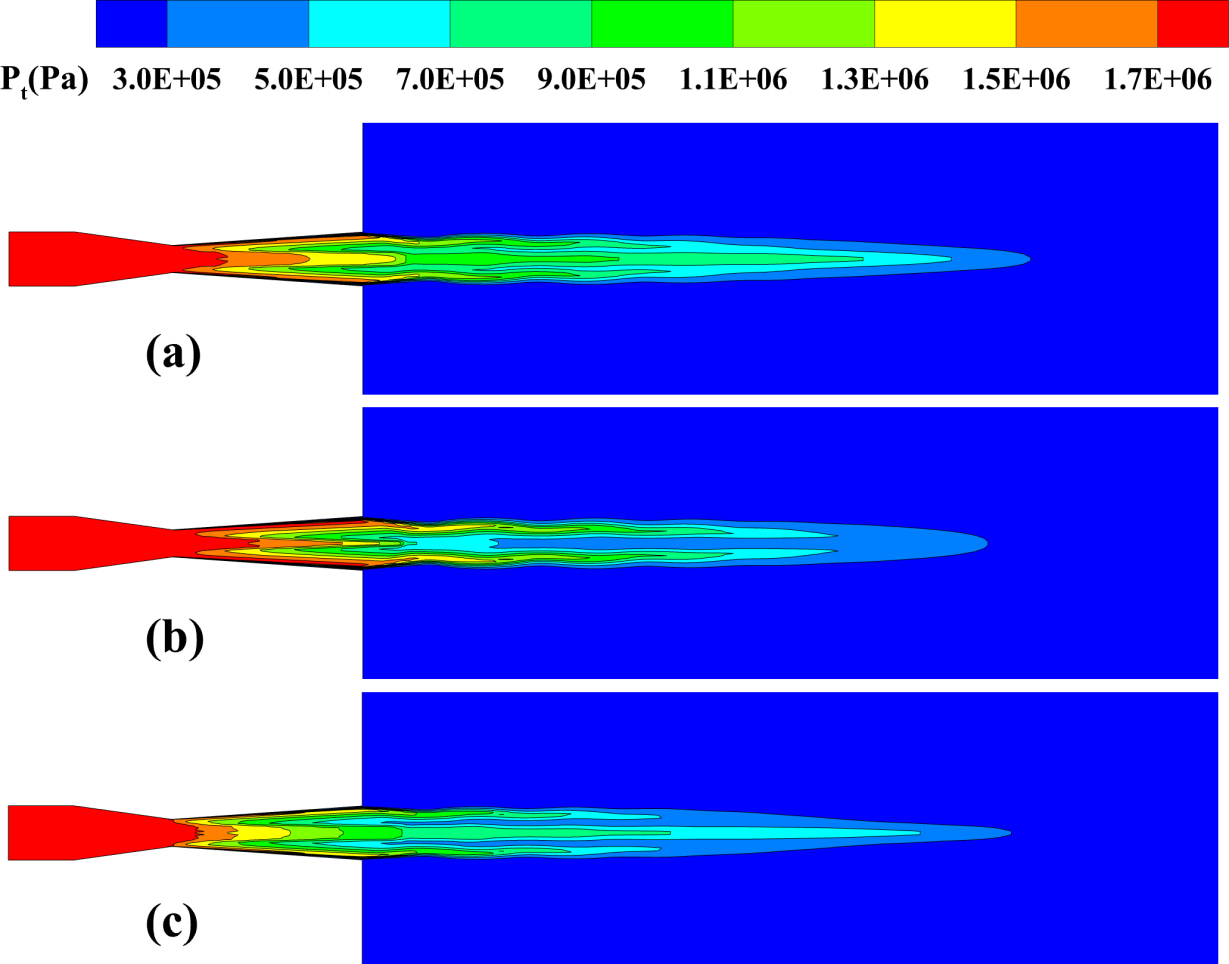
Distribution of gas total pressure on the central plane. **(a) *d***_***p***_** = 30 μm, *Q***_***p***_** = 5 × 10**^**–3**^ **kg/s; (b) *d***_***p***_** = 100 μm, *Q***_***p***_** = 5 × 10**^**–3**^ **kg/s; (c) *d***_***p***_** = 30 μm, *Q***_***p***_** = 1 × 10**^**–2**^ **kg/s.**

**Fig 13:**
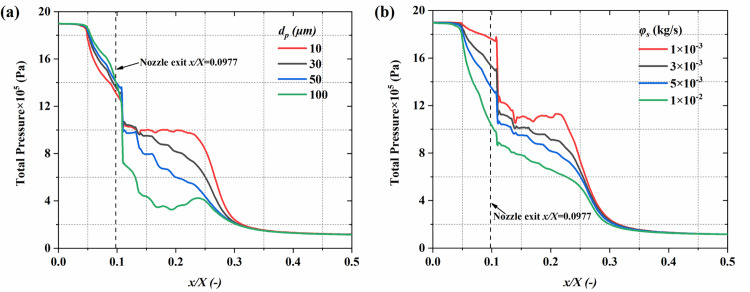
The influence of particle parameters on the time-averaged gas total pressure distribution along the system’s centerline. (a) particle diameter; (b) mass flow rate.

The total pressure, representing the overall mechanical energy of the gas, reflects the dissipation of mechanical energy into internal energy and the energy transfer characteristics between the gas and particles. Fig 12 provides the central plane gas total pressure distribution. under different particle diameter and mass flow rate conditions. Observations indicate that the total pressure reduces axially, showing a typical trend of pressure decay. Under larger particle diameter conditions, due to the particles’ enhanced disturbance of the gas flow field, the non-uniformity of the total pressure distribution is more pronounced, especially in the spray nozzle exit and downstream regions. In contrast, under high mass flow rate conditions, the significant increase in particle concentration, along with more frequent interactions between particles and gas, strengthens the momentum transfer process, further exacerbating momentum loss. This results in the low-pressure region moving closer to the nozzle outlet, and the total pressure decreases more rapidly. Fig 13 provides a quantitative analysis of how particle size and mass flow rate affect the gas total pressure distribution. The results show that larger particles significantly exacerbate the reduction in the gas’s total pressure. Specifically, at 10 μm in diameter, the peak value of total gas pressure is 1.02 MPa, whereas at 100 μm, the pressure drops to 0.62 MPa. The increase in particle resistance and enhanced energy dissipation are the main reasons for the rapid drop in total pressure. Similarly, a rise in particle mass flow rate significantly affects the total pressure distribution. From 1 × 10^-3^ kg/s to 1 × 10^-2^ kg/s, the concentration increases substantially, strengthening the momentum exchange between the gas and particles, which causes the total pressure downstream of the nozzle to decrease from 1.78 MPa to 0.85 MPa.

### 4.2 Particle properties

#### 4.2.1 Particle velocity.

**Fig 14:**
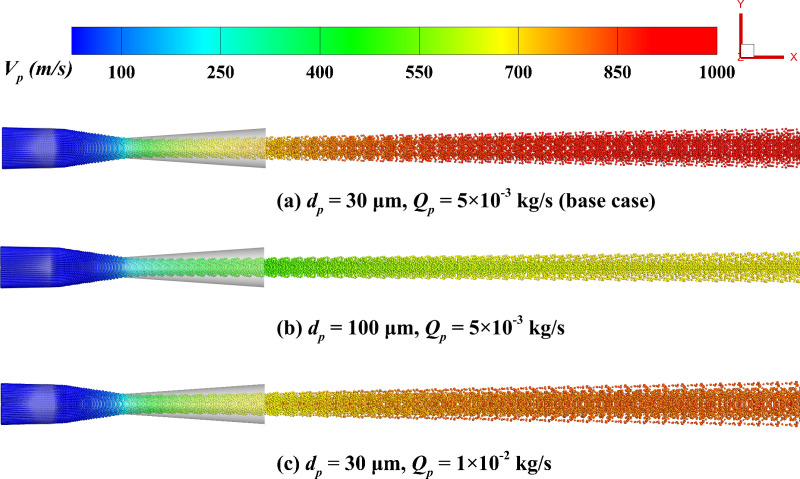
Particle distribution colored by particle velocity. **(a) *d***_***p***_** = 30 μm, *Q***_***p***_** = 5 × 10**^**-3**^ **kg/s; (b) *d***_***p***_** = 100 μm, *Q***_***p***_** = 5 × 10**^**-3**^ **kg/s; (c) *d***_***p***_** = 30 μm, *Q***_***p***_** = 1 × 10**^**-2**^ **kg/s.**

**Fig 15:**
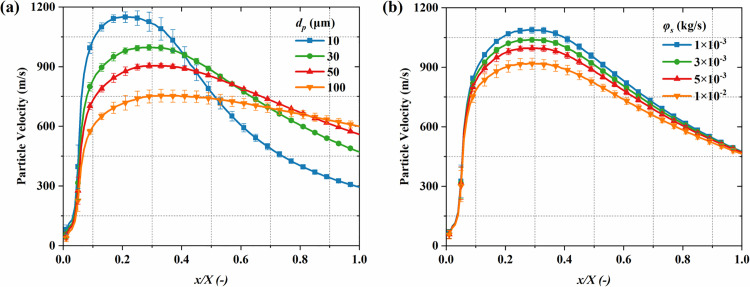
Influence of particle parameters on the time-averaged particle speed distribution along the system centerline. (a) particle diameter; (b) mass flow rate.

Fig 14 illustrates the particle velocity distribution characteristics under different particle diameters and mass flow rate conditions. The growth in particle size results in increased particle inertia, consequently slowing down the acceleration process, making it difficult for particles to quickly reach the airflow velocity, with a noticeably lower peak velocity. Larger particles, due to their stronger inertia, experience weaker radial disturbances, leading to particles being more concentrated in the main flow region and are less likely to disperse toward the edge regions. Additionally, at 1 × 10^-2^ kg/s, the significant increase in particle number results in more frequent momentum exchanges between the gas and particles, weakening the gas-induced driving force acting on the particle. Consequently, the particle peak speed decreases, and the particle distribution becomes more dispersed. Fig 15 provides a quantitative analysis of how particle size and mass flow rate affect the particle velocity distribution. The error bars in Fig 15a and 15b represent the standard deviation of particle data within spatial intervals. Each data point indicates the average value of a physical quantity (e.g., velocity) for particles within that interval. This explanation of error bars is consistent throughout the paper and will not be reiterated in subsequent sections. The results indicate that smaller particles achieve higher peak velocities, attributed to their lower inertia, enabling them to quickly adapt to changes in gas speed. Specifically, at 10 μm, the maximum particle velocity reaches 1149 m/s, whereas it decreases to 754 m/s for particles of 100 μm in size. Similarly, the rise in mass flow rate greatly suppresses the particle maximum velocity. As the particle number increases, gas phase momentum is more evenly distributed among the particles, reducing the driving force applied by the gas on individual particles, thereby limiting particle acceleration in the flow direction. Specifically, from 1 × 10^-3^ kg/s to 1 × 10^-2^ kg/s, the peak speeds are 1089 m/s and 919 m/s, respectively.

#### 4.2.2 Particle temperature.

**Fig 16:**
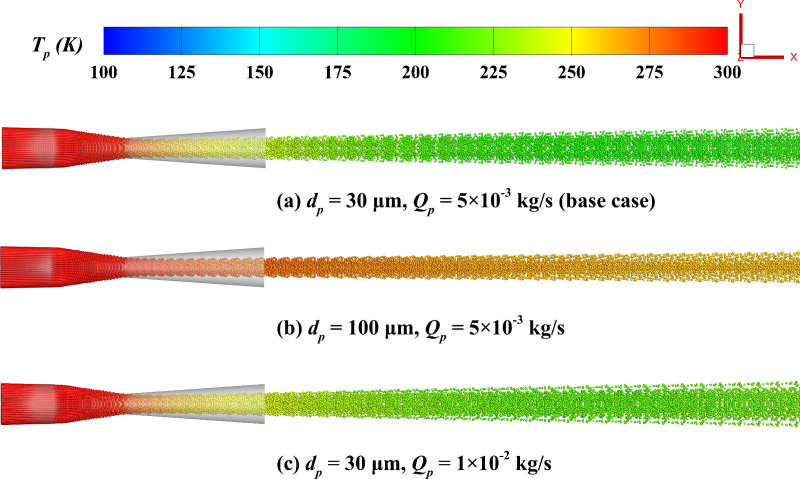
Particle distribution colored by particle temperature. **(a) *d***_***p***_** = 30 μm, *Q***_***p***_** = 5 × 10**^**-3**^ **kg/s; (b) *d***_***p***_** = 100 μm, *Q***_***p***_** = 5 × 10**^**-3**^ **kg/s; (c) *d***_***p***_** = 30 μm, *Q***_***p***_** = 1 × 10**^**-2**^ **kg/s.**

**Fig 17:**
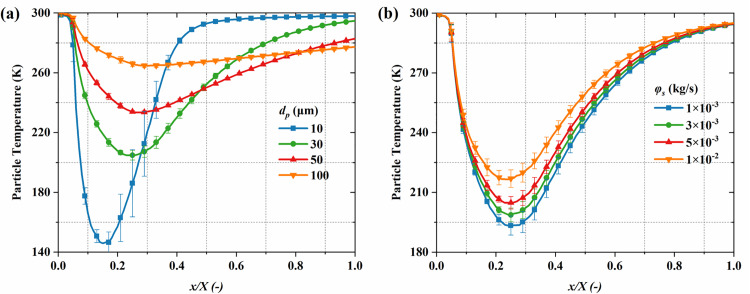
The influence of particle parameters on the time-averaged particle temperature distribution along the system’s centerline. (a) particle diameter; (b) mass flow rate.

Fig 16 illustrates the particle temperature distribution. The particle temperature rapidly decreases near the nozzle throat. This occurs because of the rapid gas expansion and cooling in the nozzle expansion section, which leads to a substantial decline in particle temperature. Compared to mass flow rate, the particle size plays a more critical role in influencing particle temperature. Although larger particles have a greater heat exchange area, the cooling process is dominated by mass effects, resulting in a slower temperature decay, thus larger particles typically have higher temperatures. Fig 17 quantitatively analyzes the influence of particle diameter and mass flow rate on the axial temperature distribution of particles. The findings show that as the axial position increases, in the short distance (*x/X* < 0.2), the intense heat transfer between particle and the gas causes the particle temperature to rapidly drop, reaching the lowest point, and then gradually increase back toward the inlet temperature. At the same time, smaller particles experience a greater temperature drop and a faster recovery. Specifically, for particle diameters of 10 μm and 100 μm, the minimum temperatures are 145 K and 264 K, respectively. Fig 17b further reveals the effect of mass flow rate on the temperature distribution. With the increase in mass flow rate, the particle temperature increases significantly. The increase in particle concentration is responsible for this phenomenon, which weakens the heat transfer between the gas and individual particles, thus suppressing the rapid temperature drop. Specifically, at 1 × 10^-2^ kg/s and 1 × 10^-3^ kg/s, the minimum temperatures are 216 K and 193 K, respectively.

### 4.3 Interphase interaction

#### 4.3.1 Particle Reynold number.

**Fig 18:**
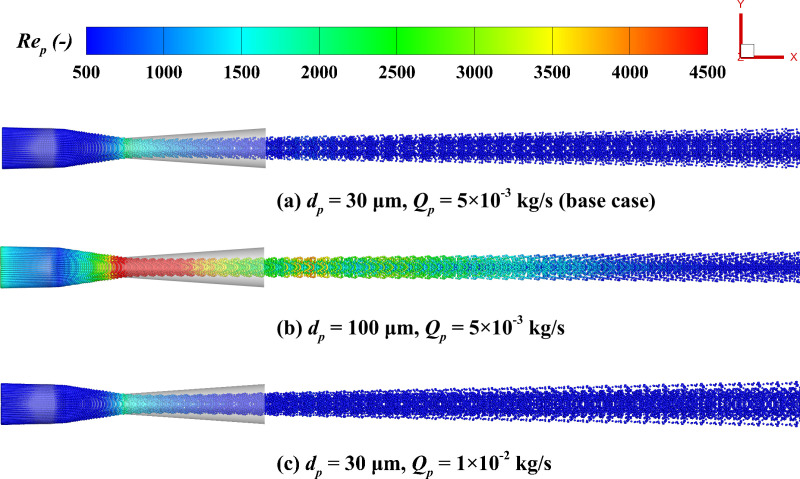
Particle distribution colored by *Re*_*p*_; (a) *d*_*p*_ **= 30** **μm, *Q***_***p***_** = 5 × 10**^**-3**^ **kg/s; (b) *d***_***p***_** = 100** **μm, *Q***_***p***_** = 5 × 10**^**-3**^ **kg/s; (c) *d***_***p***_** = 30** **μm, *Q***_***p***_** = 1 × 10**^**-2**^ **kg/s.**

**Fig 19:**
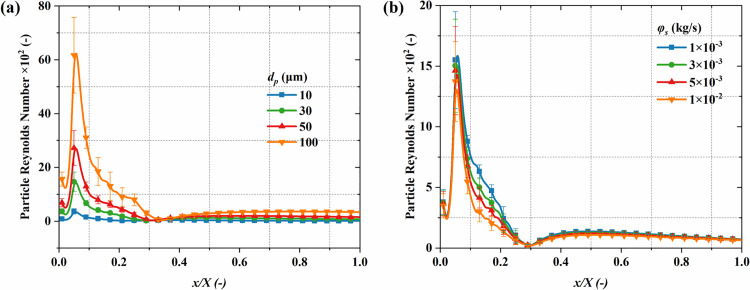
Influence of particle parameters on the time-averaged *Re*_*p*_ distribution along the system centerline. (a) particle diameter; (b) mass flow rate.

Fig 18 illustrates the distribution characteristics of *Re*_*p*_. The *Re*_*p*_ reaches its maximum value downstream of the nozzle throat and then gradually decreases and stabilizes with the rise in flow distance. This shows that the nozzle throat and its downstream region are key areas for momentum exchange in gas-particle flow, where the interaction between particles and the gas phase is most significant. Additionally, large particle diameters result in greater inertia, leading to more pronounced differences in gas velocity, thereby enhancing momentum exchange, significantly increasing the peak *Re*_*p*_, and causing its distribution to become uneven. Fig 19 quantitatively analyzes the impact of particle diameter and mass flow rate on the *Re*_*p*_ distribution along the centerline. For particle diameters of 10 μm and 100 μm, the peak *Re*_*p*_ are 367 and 6421, respectively. In the range of *x/X* > 0.4, the *Re*_*p*_ for different particle diameters stabilize and remain at lower levels, although larger particle diameters still result in slightly higher *Re*_*p*_ due to greater inertia. In contrast, the mass flow rate exhibits a limited influence on the *Re*_*p*_, as the *Re*_*p*_ is mainly influenced by particle size and the relative speed between phases, while the impact of an increase in particle number on the *Re*_*p*_ is limited. Specifically, at 1 × 10^-2^ kg/s and 1 × 10^-3^ kg/s, the peak values are 1549 and 1372, respectively, and the *Re*_*p*_ stabilizes and remains at a lower level as the axial distance increases.

#### 4.3.2 Particle Nusselt number.

**Fig 20:**
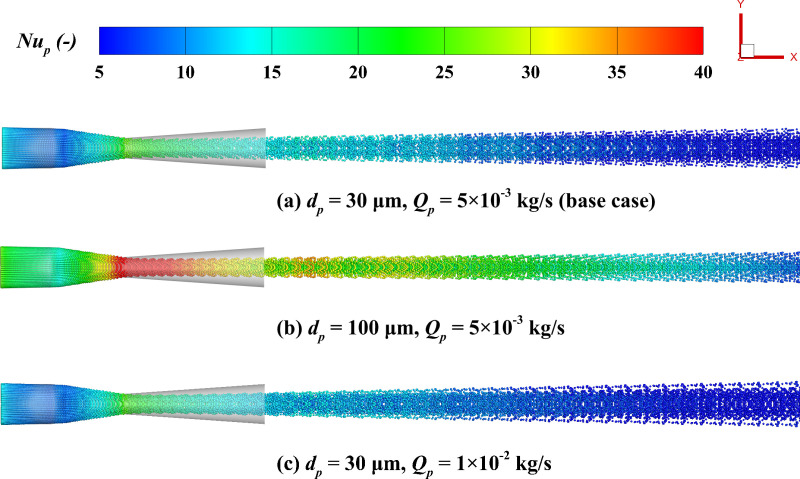
Particle distribution colored by particle Nusselt number (*Nu*_*p*_). **(a) *d***_***p***_** = 30 μm, *Q***_***p***_** = 5 × 10**^**-3**^ **kg/s; (b) *d***_***p***_** = 100 μm, *Q***_***p***_** = 5 × 10**^**-3**^ **kg/s; (c) *d***_***p***_** = 30 μm, *Q***_***p***_** = 1 × 10**^**-2**^ **kg/s.**

**Fig 21:**
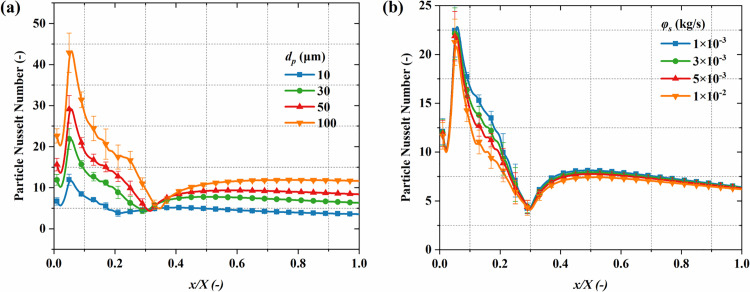
Effects of particle parameters on the time-averaged *Nu*_*p*_ distribution along the system centerline. (a) particle diameter; (b) mass flow rate.

Fig 20 demonstrates the *Nu*_*p*_ distribution. *Nu*_*p*_ peaks at the nozzle throat and then decreases gradually, eventually stabilizing as the flow progresses. This indicates that the nozzle throat is the most active region for thermal exchange in the gas-particle flow, and the thermal efficiency gradually declines as the flow distance increases. The particle diameter plays a critical role in influencing the *Nu*_*p*_. Larger particles possess a greater surface area, which enhances their heat exchange capacity with the gas, leading to a significantly higher *Nu*_*p*_ compared to smaller particles. Fig 21 quantitatively analyzes the impact of particle diameter and mass flow rate on the *Nu*_*p*_ distribution along the centerline. Larger particles exhibit higher *Nu*_*p*_, with peak values of 12 and 44 for particles of 10 μm and 100 μm in size, respectively. The mass flow rate exhibits a limited influence on the *Nu*_*p*_. At 1 × 10^-2^ kg/s and 1 × 10^-3^ kg/s, the *Nu*_*p*_ are 23.26 and 21.25, respectively, with no significant difference. This is mainly because the size of the *Nu*_*p*_ is primarily determined by the characteristic size of the particles, and the change in particle count exerts a limited influence on the heat exchange capacity of individual particles. Additionally, near *x/X* = 0.3 along the nozzle axis, the *Nu*_*p*_ shows a rebound. This happens due to the faster reduction in gas speed compared to the particles, which increases the local phase velocity difference, thereby enhancing convective heat transfer at this location. In the region of *x/X* > 0.5, the *Nu*_*p*_ stabilizes and remains at a lower level, indicating that the thermal exchange efficiency in this region approaches saturation and the heat transfer between particles and the gas gradually reaches equilibrium.

## 5 Conclusion

In this study, the Euler-Lagrange framework with the DPM was employed investigate the impacts of particle size and mass flow rate on the gas-phase compressibility, particle velocity, particle temperature, and inter-phase interactions in a Laval nozzle. Some interesting findings are outlined below:

1) In terms of the influence of material particles on the compressibility of the gas phase, larger particle sizes have a greater effect on compressible structures, while a rise in mass flow rate absorbs more energy from the gas flow field, reducing the gas expansion capacity and leading to lower velocity, Mach number, and higher temperature.
2) In terms of the influences of particle size and mass flow rate, smaller particle sizes reach the maximum velocity further upstream. This implies that in the design of a needle-free injector, the distance between the Laval nozzle exit and the skin must match the particle size of the drug particles to achieve the maximum penetration effect. Moreover, smaller particles have lower temperatures. When the particle size increases from 10 μm to 100 μm, the minimum temperatures of the particles are 145 K and 264 K, respectively, which requires matching the particle diameter with the minimum temperature at which the drug particles do not degrade. Additionally, as the drug dose increases, the injection velocity and penetration ability decrease to some extent, and the dosage range must be reasonably controlled.
3) In terms of inter-phase interactions, the momentum and energy exchange between phases occurs primarily in the core of the jet. The length of the jet core region is approximately in the range of *x/X *= 0.3. Outside the core region, the gas phase velocity quickly decays, and the temperature increases rapidly. Within the core jet region, drug particles are accelerated and cooled, while outside the core region, the particles decelerate and heat up. Strong inter-phase interactions primarily occur in the nozzle’s expansion section and the core jet region.

## Supporting information

S1 File Simulation datasets. (ZIP)
